# Parenting capacity and resources while living in temporary accommodation in England during the COVID-19 pandemic: a CHAMPIONS project qualitative investigation of raising children under 5 in resource-limited settings

**DOI:** 10.1136/bmjopen-2025-099328

**Published:** 2025-11-12

**Authors:** Monica Lakhanpaul, Nadzeya Svirydzenka, Boushra Khan-Lodhi, Marcella Ucci, Michelle Heys, Raghu Raghavan, Kriss Fearon, Matthew Ankers, Yvonne Karen Parry

**Affiliations:** 1 Population Policy and Practice Research and Teaching Department, University College London, London, UK; 2 Nottingham University Hospital NHS Trust, Nottingham, UK; 3 De Montfort University, Leicester, UK; 4 University of the West of England, Bristol, UK; 5 Institute for Environmental Design and Engineering, The Bartlett, University College London (UCL), London, UK; 6 East London NHS Foundation Trust, London, UK; 7 College of Nursing & Health Sciences, Flinders University, Adelaide, South Australia, Australia

**Keywords:** parents, caregivers, child, COVID-19, qualitative research

## Abstract

**Abstract:**

**Objective:**

In middle-income to high-income countries, temporary accommodation (TA), regardless of type, is considered a form of homelessness. Families with young children living in these countries, in these circumstances, often become disconnected from friends, family and services (such as health and welfare support). The additional impact of pandemic restrictions on parents with children under 5 already living in TA had the potential to be considerable. However, this remains an area of limited research. To address this, this study explored the experiences of parents with children under 5, who lived in TA during the pandemic.

**Design:**

The research adopted a qualitative descriptive approach, using semi-structured telephone interviews with 41 families, to explore parents’ experiences of living in TA during the pandemic, with a child under 5. Interviews considered a broad range of factors such as housing quality, access to healthcare and education and the environment. Interviews were audio-recorded, transcribed verbatim and then analysed using a thematic analysis approach.

**Results:**

Parent interviews identified that living in TA with a child under 5 during the pandemic impacted their access to services such as healthcare and ability to gather resources, while also affecting their mental health and general well-being. These parent experiences were detailed in three themes, including: (1) effect of restrictions on access, which included healthcare, environment and basic necessities; (2) impact on parents, which included mental health, physical and social impacts and (3) supports, including support services and networks.

**Conclusion:**

The challenges conveyed by COVID-19 restrictions, on those already parenting a young child while living in TA, caused concerning health impacts for those affected, while also having potential developmental side effects on their children. This indicates the urgent need for targeted interventions and policies to support vulnerable families in TA, ensuring their well-being during crises and beyond.

**STRENGTHS AND LIMITATIONS OF THIS STUDY**

- Qualitative descriptive design: this approach allowed for in-depth exploration of participants’ experiences, providing rich and detailed data.
- Semi-structured interviews: enabled flexibility in questioning, allowing participants to share their experiences openly and comprehensively.
- Thematic analysis: facilitated the identification of patterns and themes within the data that gave considerable insights into the lived experiences of parents living in TA during the pandemic.
- Remote data collection: may have limited the depth of interaction and the ability to capture non-verbal cues.
- Time and place: the study’s findings are specific to the COVID-19 pandemic period, which may limit the generalisability of the results to other times or contexts

## Introduction

 In England, temporary accommodation (TA) is used by local housing authorities to shelter unintentionally homeless families, as they have a duty to accommodate them, as set out in part 7 of the Housing Act 1996.^1^ Similarly, TA is provided as a short-term solution by support organisations to address a group or individuals’ housing crisis.^1^ Examples of TA include private rentals, social housing, hostels/refuges, bed and breakfasts or mobile homes.^1^ TA may also take the form of sofa surfing, including staying with friends/relatives, squatting, night shelters, sleeping rough on streets/in parks or various types of shared TA.^2 3^ Regardless of the type, however, it should be noted that living in TA is considered a form of homelessness, due to its inherent lack of permanency.^4^ Moreover, despite the diversity of forms that TA can take, much of the available accommodation in the UK is unsuitable for children, as they can lack safe play areas, clean toilet facilities, adequate food storage or laundry access and may be unhygienic, vermin-infested, cold or damp.^5 6^ Families living in TA also move frequently, and it is common for them to end up far removed from existing support networks at a moment’s notice.^5^ Parry^7^ notes that the use of TA for families by councils, local and/or national government is increasing in middle-income to high-income countries, with successive policies having done little to address the dearth of housing, or factors that cause homelessness. Unfortunately, the use of the word ‘temporary’ is also often a misnomer, as many households that end up in TA in England stay there for many years due to a lack of suitable permanent housing.^8^

Problematically, the ‘households in temporary accommodation (England)’ report by Wilson and Barton^1^ noted a sharp increase in the numbers of households needing TA in the second quarter of 2020, which was linked to the COVID-19 pandemic. While more recently, the number of households with young children living in TA in 2023 continues to rise, with 142 490 children reported in England alone.^9^ The experience of living in TA for adults can impact on the quality of their parenting, as it places considerable stress on them. For example, a lack of stable and secure housing impacts on parents, as they lack a sense of permanency regarding their situation, which also impacts their ability to plan for the future.^10^ Other issues include overcrowding, lack of child safe play spaces (or space to play in general), shared bathroom facilities with other residents or even accommodation with no cooking facilities.^10 11^ These stressors cause an increase in the parents’ own stress and can lead to impacts on mental health, cause isolation and affect general well-being.^10 12 13^ When the impact of TA is combined with the potential effects of the COVID-19 pandemic, such as the threat of illness, lack of connections and closed social supports/institutions due to lockdowns, these can push a parent beyond their capacity to cope.

Interestingly for children, qualitative longitudinal research has described both positive and negative experiences of living in TA during COVID-19 restrictions.^12 14^ Positive experiences included children having more time with families to bond, more time to learn new skills and more time in general as they were no longer commuting to school, due to restrictions/lockdowns.^12^ Negative experiences included having nowhere to isolate due to an inappropriate number of rooms in TA relative to a family size, and living through the same sequence of events, within the same confined space, which impacted children’s mental health.^12^ Research from the UK by Rosenthal *et al*^10^ also reported developmental delays and regression in developmental milestones in children, during the COVID-19 restrictions. Moreover, long-term exposure of children to living in TA has significant links to poorer long-term health outcomes (including both physically and mentally), lower academic achievement and an increased risk of adult homelessness.^11 15 16^ Hence, the potential additional impacts of the COVID-19 pandemic on children living in TA, including the potential effects of increased burden placed on parents and how that might flow on to the child, is worrying. This is also despite previous research in this area stating that children who are disconnected from socialisation and the social institutions normally associated with childhood and appropriate child developmental can have lifelong detrimental health and well-being outcomes.^11 15 16^

Given the noted issues for parents and children residing in TA during the COVID-19 pandemic, this paper sets out to expand current knowledge of these realities by exploring the experiences of parents with children under 5 living in TA during COVID-19. Of interest were the effects of these factors/events on the parent’s mental health, the influence of COVID-19 restrictions on parenting and the impacts on the supports that were available. Findings reported in this paper were drawn from a wider research initiative known as the Children in Homeless Accommodations Managing Poverty Invisibility Or Non-inclusive Strategies (CHAMPIONS) project (https://www.championsproject.co.uk).

## Materials and methods

### Study design

The research used a qualitative descriptive design to explore the experiences of parents with children under 5 years who lived in TA in England during the COVID-19 pandemic.^17 18^ A qualitative approach was adopted by the research, as it allows deeper, more meaningful insights into participant experiences.^19^ Data for the study were collected via semi-structured telephone interviews. This approach allowed detailed yet open data to be gathered through probing and open-ended questions.^20 21^ The Social Ecological Model^22^ theory was used as a guide in the development of the interview schedule, to help explore key social determinants such as housing quality, access to healthcare and education and the safety of the environment. In addition, the study was informed by the Healthy Child Programme, a UK government initiative designed to help identify a child’s health and well-being needs in the early years, and which recommends interventions to support parents.^23^ This initiative helped the interview schedule have an integrated health perspective regarding the questions asked, as this was an area of interest to the wider research project. Questions explored the impact of COVID-19 restrictions on the psychological, social and emotional development of the family, any positive/negative impacts of the COVID-19 pandemic and any advice interviewees would give to other parents living in TA. Finally, the interview schedule was reviewed by the project’s Community Engagement Partners Panel, consisting of families with lived experiences of TA, for accuracy.

### Participants

Recruitment adopted a purposive convenience sampling approach through external organisations, such as third sector, welfare, refugee and domestic violence support services, local councils and housing providers. Organisations shared the call for participants using their usual methods of communication for contacting homeless families (eg, through WhatsApp groups, phone calls from support workers, socially distanced visits and emails). Social media was also used for recruitment (sites such as Instagram, Twitter and paid advertisements on Facebook) as were posters and leaflets placed in key locations like food banks and/or family support charities. Once participants agreed to participate, contact details were passed on to the research team, from the various organisations, or participants contacted the research team directly. The use of multiple agencies, communication approaches and social media to recruit participants helped ensure that a diverse range of people living in TA were recruited, and their lived experiences represented in the research. This was important as we were particularly interested in representing different types of TA as well as ethnic groups and different languages. To help facilitate this, where necessary, we provided support for participation by employing translation services and providing data vouchers to cover phone calls.

Among the narratives gathered, we are confident this diversity was well represented. Nonetheless, we acknowledge the likelihood of selection bias, which is an inherent challenge in research involving transient populations. Factors such as willingness to participate, stable telephone access, language barriers and time constraints associated with caring for young children may have influenced those who were able to take part. Moreover, we were unable to calculate precise recruitment or dropout rates for two key reasons. First, we relied on third-sector organisations for recruitment and to approach families within their remit, so exact numbers of families approached are unknown. There were different waves of recruitment by the third-sector partners as well to reflect the transient nature of this group. Second, we only approached and interviewed those families that had given us permission to access them. This limitation is discussed in the manuscript as part of a broader reflection on the methodological challenges of engaging marginalised and underserved communities.

### Patient and public involvement

Parents with lived experience of TA were consulted at every stage of this project design and implementation, including the identification of research questions, co-development of the interview guide and review of the analysis. The parents shaped the interpretation of the themes regarding the access to health, housing quality and challenges facing parents of young children during the pandemic. Throughout the project, the parents provided feedback on the protocol and content of research process and materials, and were consulted on the dissemination approaches and the language used in materials to maximise inclusion and representation.

The inclusion criteria were parent(s) over the age of 18 years living in England, who had a child under 5 and who had experienced homelessness/lived in TA during the COVID-19 pandemic. Exclusion criteria included parent(s) with no child under 5 years, who lived separately from their children or who were under the age of 18 years; people who only looked after children under 5, parent(s) who did not live in England or parent(s) who had not experienced homelessness/lived in TA during the COVID-19 pandemic. Given the vulnerable nature of the cohort and the risk of COVID-19 infection, the risk to the public of participating in the research design was considered greater than the benefits and hence was not incorporated into this study. Risks for participation were not limited to physical risks, as interviews took place via the phone; they also included possible distress from sharing stress-inducing details of experiences of living in TA and any impact on child health. We had procedures in place to mediate distress if and when it occurred and also directed all participants to free resources available to them to mediate any distress from sharing their stories with us.

### Materials and procedure

Interviews were conducted remotely to respect both COVID-19 regulations and the practical difficulties of interviewing participants in living situations, which could not accommodate visitors. Participants received digital information sheets explaining the purpose of the study. However, due to many participants living in digital poverty and only having access to a phone, the ability to read long documents was compromised. Hence, key points were repeated verbally before interviews took place (including the rights to confidentiality, anonymity and the right to withdraw from the study without consequence), as well as consent forms being discussed and consent being recorded if the document could not be signed prior to the interview. Additional steps to address inclusion issues included offering participants phone credit, translation assistance and to have a community supporter present during interviews. Interviews lasted approximately 60 min and were audio-recorded and transcribed verbatim; participants received a gift voucher for their time. Data were stored in accordance with the data management plan developed in line with General Data Protection Regulations principles and approved by Data Protection services at University College London and De Montfort University.

The position of the researcher can impact the research in a number of ways. Researcher (KF), who conducted interviews, is noted as white, middle class, employed and a securely housed person, with no lived experience of homelessness, and hence an outsider to those interviewed.^24^ They were also new to the subject area, and so had no preconceived biases or orientations based on previous experience with the sample group. Moreover, as many families taking part in the research were single parents with young children who had been particularly isolated and suffering difficult experiences living in TA during COVID-19 restrictions, there was noted value in talking to someone outside of their inner formal and informal support circles. Hence, the research interview acted as a way of witnessing participants’ challenging situations, including complaints about staff, healthcare workers and accommodation, as well as their resilience, enabling the parents to feel heard and for their perspective to be valued and included. In addition, there was a noted power imbalance, due to the incentive offered to people to participate in the research. Conducting phone interviews was one way in which participants could retain some control, as the researcher was not entering their space and they could end the interview at any point. Moreover, the researchers guaranteed that participants would receive their incentive, regardless of their level of involvement. This was also a way for participants to manage their own risk of harm in taking part.

### Analytical approach

The research used an inductive, analytical approach in our thematic analysis of the data informed by the guidelines set out by Braun and Clarke.^25^ The approach, set out by Braun and Clarke,^25^ provided the flexibility and in-depth approach required to identify all important themes within the dataset. Thematic analysis achieves this by facilitating the identification of patterns, similarities and differences within a large dataset and allows interpretations to be made on these findings.^18 26^ Additionally, it helps summarise key themes, which provide profound insight into the experiences, feelings and thoughts of individuals.^20 25^ A ‘coding tree’^27^ was created in NVivo, with input from members of the research team, which helped classify and refine themes and establish the inter-relationships between them. These combined steps produced a rich and comprehensive account of participants’ experiences, thoughts and feelings.

## Results

In total, 41 participants were interviewed in England during a 9-month period (May 2021–January 2022). The sample consisted of 39 females and two males, with an age range of 20–45 years. Various types of TA were reported, including sofa surfing, shared accommodation (like a refuge or hostel), hotel rooms or a temporary house/flat to list a few of the examples. Participants came from across England, including Bexley—one, Bradford—one, Bromley—one, East Midlands—one, Essex—two, Greater Manchester—one, Leicester—eight, Leicestershire—one, London—10, North London—one, Nottingham—three, Southeast based—one, West London—one, West Yorkshire—three (declined to disclose—six).

Table 1 shows demographic details regarding the research cohorts.

**Table 1:** Demographic data

Participant ID	Age range (years)	Gender	Type of TA
**P1**	30–35	Female	Flat
**P2**	25–30	Female	Shared accommodation
**P3**	25–30	Female	Shared accommodation and then flat
**P4**	20–25	Female	Studio flat and then shared accommodation
**P5**	25–30	Female	Hotel and then flat
**P6**	25–30	Female	Flat
**P7**	30–35	Female	Refuge
**P8**	35–40	Female	N/A
**P9**	30–35	Female	Flat
**P10**	30–35	Female	Shared accommodation
**P11**	30–35	Female	Shared accommodation
**P12**	35–40	Female	N/A
**P13**	20–25	Female	Refuge
**P14**	20–25	Female	Shared accommodation
**P15**	35–40	Female	Shared accommodation
**P16**	30–35	Male	House
**P17**	30–35	Female	Flat
**P18**	20–25	Female	Flat
**P19**	30–35	Female	Flat
**P20**	35–40	Male	House
**P21**	40–45	Female	House
**P22**	20–25	Female	Shared accommodation
**P23**	25–30	Female	House
**P24**	20–25	Female	Refuge
**P25**	40–45	Female	Flat
**P26**	35–40	Female	Shared accommodation
**P27**	25–30	Female	Refuge
**P28**	35–40	Female	N/A
**P29**	25–30	Female	N/A
**P30**	25–30	Female	Studio flat
**P31**	35–40	Female	Shared accommodation
**P32**	25–30	Female	Shared accommodation
**P33**	35–40	Female	Flat
**P34**	20–25	Female	Shared accommodation
**P35**	40–45	Female	N/A
**P36**	25–30	Female	Shared accommodation
**P37**	35–40	Female	Studio flat
**P38**	30–35	Female	Hotel
**P39**	35–40	Female	Sofa surfing
**P40**	35–40	Female	Shared accommodation
**P41**	30–35	Female	Flat

N/A, not available.

Overall, the interviews identified that living in TA with a child under 5 during the COVID-19 pandemic brought many challenges and adaptations, the details for which are outlined in three main themes:

1. Effect of restrictions on access, which included healthcare, environment and basic necessities.
2. Impact on parents, which included mental health, physical and social impacts.
3. Supports, including support services and support networks.

### Theme 1: effect of restrictions on access

COVID-19 restrictions impaired participants’ access to resources that were already compromised from living in TA. Specifically, many participants described restricted access to healthcare, their environment and basic necessities, and also discussed how these restrictions affected them. It should be noted that while some of the restrictions on people’s access came from pandemic-related causes, others are a direct result of living in TA and are discussed as such below, when relevant.

#### Healthcare restrictions

Participants described experiencing a lack of healthcare support from general practitioners (GPs), dentists and similar professionals as a result of pandemic restrictions. The lack of support was exacerbated by certain experiences like having to wait on hold, only to be told no appointments were available, as P5 notes:

Right yeah just making an appointment, you’re on the phone for like 15, 20 minutes and then when you get through it’s like sorry all the appointments are taken. And then quite a few appointments will happen on the phone.

Or exacerbated by gaining an appointment that was delivered in a format deemed inappropriate to the child’s health needs:

One of his appointments regarding his breathing was done via video call, which I found so shocking because you need to hear his chest…it’s definitely affected the way that we got appointments and the way that he was seen by GPs and other care professionals. (P5)

However, as participant P4 highlights, an inability to gain a medical appointment was not an exclusive consequence of the pandemic, but rather could also be an effect of living in TA:

…because I moved around quite a lot [due to TA], different areas have different GPs so I’ve changed GPs quite a lot. So yeah, it was quite hard with notes getting passed on and sometimes people wouldn’t understand what was wrong. And then the notes would come through a couple of weeks later and things like that.

Although the quotes are expressed by different participants and concern different impacting variables, both factors affected access to resources. These, in turn, worked against the parent and their efforts to keep their child healthy and safe (and it is more than likely that one, helped make the impact of the other worse).

#### Environment restrictions

Participants discussed experiencing restrictions on their environment such as their visitors not being allowed into their accommodation due to the rules established by those providing the TA, to limit the potential spread of COVID-19. These restrictions appeared to be in addition to the normal pandemic restrictions as well and worked to cut participants off from their support base. For example, P1 discussed how:

There was a part where I couldn’t go for antenatal classes so there was not much of that experience for myself, and there was no postnatal gatherings either. I wasn’t meeting all the mums, the people that could be my support network. I had no access to them just because the accommodation won’t let people come through because it was like a hostel kind of setting. So, it just makes it difficult in terms of me being able to have contact with people.

Participants also discussed being cut-off from their support base due to travel restrictions, pandemic-related impacts to travel services and/or having moved away from their supports due to TA status. For example, P15 stated:

…I was living close to family and friends, you know, my friends back where I was living… We go to their place, we will play, we have play dates and all that. But, because I’m far from them [now], like I said, I might go out and I’m coming back, now the train will say no train, the stress only can kill.

Regarding the effects of pandemic restrictions more generally (note, not TA-related), a number of participants identified the positives of still being able to go for a walk in the park, and noted the negatives of their children not being allowed to access the playground. When coupled with living in a small, temporary space, these limitations impacted parents, especially those with children with additional needs. For example:

…we were allowed to go for walks in the park, but not for my children to attend the playground…So most of the time we’re confined in our small room. It was one of the biggest challenges of my life…my son suffers from autism, and they need structure they need routine. And obviously, his routine changed during the pandemic. And I had to, obviously there were negative effects in terms of his behaviour, and lots of other things. (P26).

Similarly, participants noted that existing in a small space due to TA, and being restricted to that space due to COVID-19 restrictions, limited the space their child could play in and, moreover, because the family’s many belongings were compacted into that small space, the area potentially became unsafe:

There’s so many things here that are not child proof, which means I am constantly on my feet when she’s moving around to make sure that she doesn’t bump her head or pull something down or you know follow her. (P1)

Existing in, and being restricted to, these small spaces because of the unsuitable nature of many TAs, and the pandemic restrictions, also had the potential to limit a child’s physical development, as P38 highlights:

I could see he wanted to start crawling. But now what will I do I don’t have space in my room to put him on the floor, I managed but I had a tiny, tiny space in my room …because he was just starting…it was ok for him. But then later on when he knew how to crawl properly that space wasn’t enough for him anymore, because he now wanted to go everywhere, and the room is small.

Overall, many participants found that their TAs were not sufficient in terms of space for them to live properly, which meant children were unable to move around and explore. While limited access to the outdoors due to COVID-19 restrictions impacted participants’ ability to provide an alternative child-safe place, all of which had the potential to affect the child’s normal development.

#### Restricted access to necessities

Due to COVID-19 restrictions, many participants stated that they lacked access to basic necessities such as food, since grocery delivery slots, designed to help vulnerable families access food, were inappropriately timed or just did not exist. The alternative of going to the grocery store meant people risked contracting COVID-19, which for participants like P5 was problematic:

…there were services out there for the vulnerable, but they were really hard to gain access to, like for example, supermarket slots. They said they made them available to people with children…but every supermarket I was calling was the same, they said, things like oh, our slots are taken. I didn’t get a slot the whole of lockdown…They would say, oh, you can get one next week. And I was like, I need milk today not next week…then I’ll just have to get myself together and go and get myself some milk, but it was tough, because of my asthma I can’t wear a mask. It was crazy, so I’m walking into Tesco looking at everyone thinking oh my god, someone’s gonna have COVID someone’s going to give it to me.

Even venturing to the store for groceries was no guarantee for participants to gain access to the food they wanted/needed, as many shelves were empty and lacking food:

…they’re times you go and the shelf is practically empty, what do you do. Your child will need to feed. (P1)

In addition to limited access to food, participants also noted restrictions in their TA facilities, such as not being allowed to use the dryer:

…they took the utility room away from us and we weren’t allowed to use the dryer. And we weren’t allowed to put clothes on the radiators because it would make the radiators rusty…We each had a clothes airer in our bedroom through the winter that was just not fun. If you had a big load of washing you couldn’t do it, you had to do it in smaller loads and it took more time, more powder, more everything, and then it wouldn’t always dry very quickly either. (P2)

Or having limited access to basic entertainment like television:

We were sort of restricted on what we could watch on the television, we only really had one or two channels…we were stuck with terrestrial telly and couldn’t really watch anything else. So, if there was nothing on to watch it was just sit twiddling your thumbs kind of thing. (P7)

Which potentially further impacted people’s ability to exist in TA while being restricted to their accommodation due to the COVID-19 pandemic.

### Theme 2: impact on parent

This theme explores the effect of the pandemic on parents while living in TA with a child under 5, including the mental health, physical and social impacts.

#### Mental health

Many parents self-reported impacts on their mental health due to the COVID-19 restrictions, some of which were initially tied to the fear of contracting COVID-19 and its unknown outcomes, combined with the constant messaging to stay indoors. For example, P4 noted:

at the beginning I was constantly watching it [ the news ] because I was so paranoid of catching COVID…I was constantly watching Boris [ Johnson ] …I just didn’t go out, and I think it affected my anxiety more than anything, because even to this day I still don’t really like going out.

COVID-19 restrictions also exacerbated existing mental health issues, as participant P8 noted:

P8: I’ve got PTSD.

Researcher: …has that changed during the pandemic?

P8: It was really worse during the pandemic, because I felt isolated as well…it was too much, it was very frustrating.

While another participant discussed how the TA they had been placed in was inappropriate for their mental health:

Researcher: So, you were placed in this accommodation as an emergency, because of domestic violence?

P28: Exactly, yeah. At first, I was in a hotel, and then they moved me here in this place.

Researcher: So, what’s your experience been of living in the accommodation you’re in, during COVID?

P28: Oh, nothing good. Nothing good…I live with fear with all these things that are happening to me with mental health… and people here are always arguing and someone there is always drunk, and the other ones are smoking things that they shouldn’t…

The COVID-19 restrictions, living in TA or a combination of both, all had the potential to impact participants’ mental health, including exacerbating existing issues.

#### Physical impacts

A common physical impact noted by participants was a lack of sleep, which arguably was more a consequence of inappropriate TA than being necessarily caused by the COVID-19 restrictions. For example, P17 explains the reality of having one bed and all of her children in there with her:

I don’t think it affects the kids sleep but it does affect mine, because there’s all three of them sleeping right next to me, and I can’t move, so I can’t sleep, I can’t get comfortable.

Another common physical impact noted by participants related to the COVID-19 restrictions was people gaining weight from a lack of movement, as P5 notes:

I gained quite a bit of weight as well during COVID…I’ve gained 6 kilos in the year…I’ve tried to like exercise and stuff during COVID at home indoors. But I feel like when you couldn’t even go out for walks, I am not going to able to lose the weight.

The physical impact of gaining weight linked to the pandemic restrictions can also potentially be linked to inappropriate TA, as it often has limited space to move round in, as similarly discussed above when children had limited space to move around in, and grow. In contrast to weight gain, participants noted not eating, due to stress or similar:

…I can’t say that I’m eating that much. I used to eat a lot better than I do now, but I know that a lot of that’s stress and things like that, anyway. It’s just like eating is never like the top priority. (P14)

Participants also noted that eating and sleeping was not a priority for them, as they were more focused on providing (and feeding) their child healthy meals and making sure they slept well.

#### Social impact

Many participants noted feeling isolated due to the pandemic restrictions, as P21 noted:

…socially, when we weren’t allowed to see our friends, or come together, we were isolated, we felt isolated.

Similarly, participants discussed their children becoming upset due to not being able to play with friends:

So, our family and friends, it affects a lot, because sometimes, they cry, they ask me to go and to their friend’s house, so I say, no, we are not allowed to go…they were upset, because of the situation, the COVID situation. (P6)

Participants also expressed concerns about their children not getting to know other family members due to the restrictions:

He’s just not really met my grandad and things, my grandads not really met my son, my son is 18 months old now. My grandma still hasn’t even met my son, because just before COVID kicked off anyway she just couldn’t see him, but then yeah like, loads of his aunties and uncles. (P14)

Restrictions meant a lack of socialisation for both parents and children with extended family and peers. Unfortunately, however, this is also a characteristic of living in TA, where frequent moves (which often occur suddenly) pull families and children from their support networks for extended periods of time. Moreover, as noted in the quote, this situation was exacerbated during the pandemic, as families could also not travel, meaning some newborns were not introduced to their extended families for considerable periods.

### Theme 3: support

This theme explores the support participants received during the pandemic while living in TA. There were mixed findings here with many participants providing examples of beneficial support from both formal and informal networks, while others described a lack of support.

#### Support network

The importance of receiving support from informal networks such as family and friends was emphasised by many participants and included practical support like meal and basic supplies:

I wasn’t cooking, I wasn’t doing anything other and my mum, my brother, would drop off food to the door. (P5)

Or less tangible supports like the presence of family being close by:

I do have family, my mum lives round the corner luckily. I have got my dad, I have got my brother, I have got three brothers but one is small. They are a big support to us. (P9)

Parents also found comfort and support in those around them. For example, P21 explained how:

I need to talk, to share my feelings, because, at the same time, my life has completely changed. I found my children, near me as a friend, because I was able to talk to them, and spend most of time with them, and they made me, think less about my situation.

Hence, having a support network during the pandemic while living in TA appeared to be beneficial for participants, providing for unmet needs and offering the extra resilience needed to work through emotional and stressful situations.

#### Support services

Participants discussed receiving support from more formal services such as charities, health visitors, the food bank and hospitals. These formal supports were perceived as beneficial in helping participants care for their child, receive medical care and in providing essential provisions such as food and clothing. For example, P3 outlined the many items they received that helped with, including:

… [the] charity that supports migrants…she helped me giving clothes and everything, nappies, bath linen…and food as well…they did bring a basket at home, in front of the door…

Participants also noted receiving assistance with food that, as noted earlier, was often hard to access:

The council were really, really helpful, they were sending like food packs, I think it was you know weekly or bi-weekly. That was very helpful, I think that was the most helpful thing during the whole pandemic. (P5)

Many participants also noted receiving assistance with their own, and their children’s, health from health visitors:

Yeah, yeah the health visitor comes to check on the baby and so we have some referrals at least, like we’re referred to, like help with our mental health and just to support us you know, to check the baby as well. (P40)

These services helped participants maintain the course, while coping with the twin impacts of living in TA and dealing with the COVID-19 pandemic, all while parenting a child under 5.

Despite the many positive examples of support services helping participants, there were a few noted limitations, one example given by P11 notes the inability of the services themselves to help:

The refuge staff, I would say, they were not being very helpful, because they could not really do anything…apart from just, you know, often ask as if I’m doing fine, if I just, just that.

While P1 noted a lack of information/communication from their health visitor:

In terms of…the health visitors…they weren’t quite helpful because communication wise, they’re not, I don’t know what to expect of them basically. It’s like I’m chasing them to know…when is the next visit, when am I supposed to expect to hear back from you.

A similar sentiment to the above quotes was echoed in interviews from other participants; participant P9 provided a reason for the noted shortcomings:

We went to the health visitor with my concerns, got an appointment, assessment, but I’m still waiting for the sessions. I have been chasing them, but I haven’t heard anything back as yet. Because of the whole COVID thing they said they’ve got so much to catch up on and all the rest of it.

Hence, the COVID-19 pandemic had impacted the health service, which caused delays in their follow-up. Thankfully, participants were, for the majority, positive about their health visitors and the help they provided.

## Discussion

This is one of only a handful of qualitative studies examining the impact of living through the COVID-19 restrictions in England, based on the experiences of parents with children under 5 who also lived in TA during this period.^10 12^ Thus, this study offers critical and unique insights into the impact of such events on these marginalised populations. Analysis of data from 41 geographically dispersed participants, of whom 39 were mothers, revealed key insights into: the increased limitations on their access to resources, the effect on their mental health and well-being, and experiences with formal and informal supports. On the topic of mental health, a number of parents in our interviews noted how the pandemic impacted on theirs, while others discussed how it exacerbated their existing issues. Many participants also described the psychological impact of pandemic restrictions on their well-being and/or having to find a balance between their own mental health needs and the needs of their child. Their ability to manage both was further impacted by scarcity of resources during restrictions and living in TA. Of note, it is hard to separate the impact of living in TA in this instance from the impact of the pandemic as, for example, Rosenthal *et al*^10^ noted similar impacts to parents’ mental health from living in TA. These impacts are connected to the transient nature of living in TA (eg, having to move frequently), which causes considerable uncertainty about all aspects of a person’s life.^10^ As Taylor and Edwards^16^ note, “secure housing tenure gives people a sense of autonomy, certainty and control that leads to lower levels of stress and increases residential stability”. This is even more important when children are involved.^10 12 14^ However, what can be said is that the pandemic restrictions, in addition to living in TA, in no way improved circumstances for these families and may have even pushed some beyond their ability to cope.

Participants also experienced a lack of support and found communication difficulties when attempting to access medical care and provisions. This increased distress and left a sense of isolation from services, including from both external and internal support providers.^5 10 12 14^ Similarly, the inability to receive visitors or to travel to see friends and family due to living in different areas or being far away (cost of travel) impacted on parents with young children. The restrictions from both the pandemic and participant TA impacted participants travelling freely to other places and having normal social contact with other households. This highlights the importance of family support, a wider support network and others who can help during stressful situations by providing provisions, financial support and emotional support.^10 12 14^ This, in turn, can be beneficial to participants’ emotional and social health by reducing stress and pressure of living alone with a child that, as the responses in this study indicate, were solely tested during the pandemic and highlighted areas that need considerable attention. Results also highlighted that access to services such as healthcare providers was impacted by both the pandemic restrictions and living in TA. Although the majority of impacts of the pandemic have now passed, the effect of moving between different jurisdictions due to living in TA still causes issues for people trying to access services, due to the siloing of services between areas. For example, due to the relocation of a family from one TA to another, one participant experienced delays in medical treatment as their information was not transferred in time between treatment facilities. This was caused by the participant living in TA and having to move between areas due to not having a permanent place to settle. The delay in information being passed on, in turn, delayed treatment for the participant, as medical practitioners in the new areas were unaware of the patient’s treatment needs. Hence, care is delayed due to having to reassess patients from scratch, or from having to wait for records from a previous GP to be passed on to the new one.

More broadly, the findings speak to the common challenges experienced by parents raising children in resource-poor environments. While the TA context of this paper might be specific to the UK,^1^ the challenges faced by parents while trying to raise and develop their child—such as mediating a plethora of environmental issues like mould, vermin, access challenges to key services, digital poverty, overcrowding and frequent forced relocations—are common for people living in homelessness, TA and even in poverty, globally, including in other middle-income to high-income countries.^13 28^ The impact of these challenges on parents themselves has universal outcomes, with consideration to their mental and physical health.^10 29^ These, in turn, impact on the parent’s ability to support the needs of their children which, in such environments, are likely to be varied and exacerbated as well, with evidence pointing to significant increases in mental health, developmental and physical health needs.^1012^,^14 30^ From a policy context, providing visibility and adequate guidelines for mediating negative impacts of homelessness and TA on parents and children (which unfortunately are often lacking) requires an urgent and immediate response.^28^ Indeed, the COVID-19 pandemic introduced new challenges to families raising young children in TA, with closures to playgrounds and community centres and moving primary care into online or phone-based appointments. Some of these unique challenges are not relevant today, with playgrounds and community centres regaining their usual operations. However, some of the modifications in primary care are still faced by families today (like phone-based appointments). A further point of intersection between the pandemic TA context and today is the fact of its unsuitability for child health and development.^5 6^ While the pandemic exposed children to this environment for a considerably longer time due to lockdowns, children are still being placed in accommodation that is damp, unhygienic, overcrowded and too cold.^5 6^ Therefore, the negative impact of unsuitable housing on children and their parents has not disappeared with the pandemic and remains a present challenge for around 150 000 families across the UK.

### Limitations

While we recognise that the data in this paper are from 2021 and are a few years old now, we believe the results still hold relevance to present day context. For example, in the 5 years since the onset of the COVID-19 pandemic, homelessness rates for families with children continue to be on the rise, with research consistently pointing to unsuitability of TA for supporting health and well-being of children, one key determinant being the well-being and parenting capacity of their parents.^5 6 10^ In addition, interviews were conducted remotely, which may have limited the depth of interaction and the ability to capture non-verbal cues. To help address this, clarification was sought when responses were unclear; remote interviews also helped protect those involved with the research from potential contraction of COVID-19. In addition, while some of this study’s findings could be recognisable to those living in TA in non-pandemic circumstances, the results are specific to the COVID-19 pandemic period, which may also limit their generalisability to other times or contexts.

Finally, while we have focused on maximising inclusivity within our recruitment strategy across England to represent diversity in TA types, ethnic backgrounds and languages spoken, we recognise that selection bias may still have influenced our findings. Families who participated were more likely to have had consistent telephone access, sufficient time to engage and, in some cases, more support from close relatives or third-sector organisations. Therefore, more vulnerable families who were experiencing acute instability, language barriers that prevented them from reading or understanding recruitment materials, legal or safeguarding fears or reluctance to engage with research may be under-represented. Such recruitment challenges and resultant biases are well-documented in studies involving transient populations^31^ and are particularly salient in research with families experiencing homelessness in the UK. These constraints should be considered when interpreting the transferability of our findings.

### Future research and impact

Future research should consider three key avenues. First, it is important to contextualise these findings within specific cultural groups and migration journeys, as these families are likely to share traumatic experiences unrelated to TA. They also come with the wealth of cultural knowledge that can be harnessed in support of their parenting journeys in the UK. Second, children with complex needs also require focused exploration regarding parenting support and environmental risk in TA. Finally, exploring specific domains of parenting in more detail would uncover more learning within areas of sleep, nutrition, education and so forth.

To support parents’ capacity to parent their children in resource-limited settings like TA in England, it is important to address three key limitations. First, child-centred accommodation is crucial to provide the environment within which children can thrive, and parents can support their children’s health and development. This means accommodation that is equipped with sufficient laundry, cooking and food storage facilities, hygienic bathroom and toilet, capacity to cool and heat the space, absence of mould, space to crawl and walk, play space indoors and outdoors to name a few. Second, families need to be supported by an interconnected network of services (health, education, housing, social care). Siloed care leads to re-traumatisation of parents and many families falling through the cracks of a disjointed system. Finally, families need to be placed within networks of support, be that their existing networks, with specific help from link workers and health visitors who reach out to these families, or within accommodation with families (and not singles) that can provide information and help.

## Data Availability

Data are available on reasonable request.
