# Supplementary material for: Parenting capacity and resources while living in temporary accommodation in England during the COVID-19 pandemic: a CHAMPIONS project qualitative investigation of raising children under 5 in resource-limited settings
Source: BMJ Open. 2025 Nov 12;15(11):e099328. doi: 10.1136/bmjopen-2025-099328 (PMC12612732; doi:10.1136/bmjopen-2025-099328)
Supplement: Supplementary file 1 [file bmjopen-2025-099328.reviewer_comments.pdf]

PEER REVIEW HISTORY

BMJ Open publishes all reviews undertaken for accepted manuscripts. Reviewers are asked to complete a checklist review form and are provided with free text boxes to elaborate on their assessment. These free text comments are reproduced below.

ARTICLE DETAILS

Title (Provisional)

Parenting capacity and resources whilst living in temporary accommodation in England during the COVID-19 pandemic: A CHAMPIONS project qualitative investigation of raising children under 5 in resource limited settings.

Authors

Lakhanpaul, Monica; Svirydzenka, Nadzeya; Khan-Lodhi, Boushra; Ucci, Marcella; Heys, Michelle; Raghavan, Raghu; Fearon, Kriss; Ankers, Matthew; Parry, Yvonne Karen

---

VERSION 1 - REVIEW

**Reviewer**	**1**
**Name**	**Schindler-Ruwisch, Jennifer**
**Affiliation**	**Fairfield University**
**Date**	**23-Jul-2025**
**COI**	

I appreciated the opportunity to read and review, “Parenting a child under 5 during the COVID-19 pandemic whilst living in temporary accommodation in England: A CHAMPIONS project qualitative investigation.” The paper has a niche objective and uses an exploratory qualitative approach to address the under-studied research area.

Introduction-

Is it possible to describe TA in England in more detail. It seems it can be very broad ranging which could impact the outcomes more substantially. Readers from outside the geographic area might like additional context or literature if available.

Methods-

Is a copy of the interview guide available (as an appendix or table)? It would be helpful to see how the Social Ecological Model and social determinants of health informed the questions more explicitly.

Given the recruitment strategy, it seems there is a strong possibility of selection bias based on a variety of factors including willingness to participate, stable phone line, language

spoken at home. Could you clarify eligibility characteristics, anyone who declined or was unreachable and then further address this bias in the discussion section?

You mention the risk of participation, but weren't all interviews done via phone? Please clarify.

COVID-19 should be consistently capitalized throughout.

For your analytical approach, was it inductive or deductive? You say data were analyzed thematically- was any particular qualitative approach used for this? Phenomenology, grounded theory etc? Please define and clarify with literature where possible.

Can you comment on the interviewers positionality and how that may have impacted the responses? In other words, where the lived experiences between the interviewers and interviewees very different and how might that have impacted the rapport and responses.

The results of this study are at least 5 years old now. How can you relate these findings to long-term impacts or areas for future planning, rather than just historical data? In other words, is it possible to add more about its relevance in present-day.

Some of the results are specific to the temporary accommodations and some are specific to more general COVID restrictions. Is there a way you could separate these out and highlight these nuances a bit more? For example, one participant notes her child could not use the playground- I assume that is a COVID restriction, not a housing related barrier? Can you elaborate more on how these may intersect?

Discussion-

Can you clarify or re-word this sentence-it's meaning is unclear to me: "For example, from the results of this research, a participant experienced delays in treatment due to a delayed in medical

information being passed on, between different area"

More literature is needed in the discussion section to tie these findings back to current evidence.

Limitations-

While you note the limitations of this work, it would be helpful to see areas for future direction to continue and complement these findings as noted above.

**Reviewer**	**2**
**Name**	**Wiwattanapantuwong, Juthatip**
**Affiliation**	**Chulalongkorn University**
**Date**	**25-Jul-2025**
**COI**	

I've read the paper entitled 'Parenting a child under 5 during the COVID-19 pandemic whilst living in temporary accommodation in England: A CHAMPIONS project qualitative investigation. The paper qualitatively explored experienced of 41 parents with child age until 5 years old using phone interview. I found this paper reflect how COVID-19 worsening life of parents with small children who had lived in temporary housing, their mental health, well-being, quality of lives gets even worse during the pandemic which are clearly supported by the study results. Additionally, I have a couple of points that, I think, need clarifications or revisions.

1. Title: While the title begins with ‘parenting a child under 5’, the content of the result reflects more on parent’s life, their community, their living, the support they obtained. I found this a bit ambiguity between the title, objective, and the results. Not any parts in the literature review mentioned about parenting children under 5. I would recommend the authors to modify the title such as, ‘Experience of living in Temporary accommodation among parents of a child under 5 during the COVID-19 pandemic in England: A CHAMPIONS project qualitative investigation.’

2. Method: If possible, please provide response rate of the contacted and completed participants, and example of questions asked to participants (are they structured or semi-structured interview?)

3. Result: The results revealed 4 themes; access to service, mental health and well-being, and support service and network.

a. Some themes are overlapping with each other. I would recommend the author to revise the selected extracts those represent the theme clearly, or using ‘bold’ font to emphasize some keywords.

b. With the combination of ‘being a parent’, ‘living in TA’ and ‘during COVID-19’ which is very unique. This current version, for me, some extract represents common experience of covid-19 (such as environmental restriction issues that most people experiencing this too, and social support), which I think will be better if the extracts has been revised.

4. Discussion: To me, the discussion looks like a summary of the result, and also, not much mentioned about parenting children under 5. It would provide more insights if the authors pick up some key issues and discuss in depth which links to key messages of the paper.

5. Implication and impact: The results show strong evidence of how parents living in TA were suffered from COVID19. I would love to read some sentences from the authors suggesting future directions or strategies to improve quality of live in TA in England.

I’m willing to read the revised manuscript and looking forward to learning more from this paper. Thank you.

VERSION 1 - AUTHOR RESPONSE

**Reviewers' comments**	
**Introduction**	
Is it possible to describe TA in England in more detail. It seems it can be very broad ranging which could impact the outcomes more substantially. Readers from outside the geographic area might like additional context or literature if available.	Thank you for the suggestion, we have added this sentence, in the first paragraph of the introduction, to aid clarity – 'Moreover, despite the diversity of forms that TA can take, much of the available accommodation in the UK, is unsuitable for children, as they can lack safe play areas, clean toilet facilities, adequate food storage or laundry access, and may be unhygienic, vermin-infested, cold, or damp (Shelter 2025; Crisis 2024). Families living in TA, also move frequently, and it is common for them to end up far removed from existing support networks at a moment's notice (Shelter 2025).'<br><br>To give more context to TA in a UK setting, we have also added these references -<br><br>Crisis (2024). Number of children in temporary accommodation up 15% in England, reaching record high. https://www.crisis.org.uk/about-us/crisis-media-centre/number-of-children-in-temporary-accommodation-up-15-in-england-reaching-record-high/<br><br>Shelter (2025). Latest government homelessness statistics: Record-high numbers of children growing up homeless in temporary accommodation. https://england.shelter.org.uk/professional_resources/policy_and_research/policy_library/latest_government_homelessness_statistics_record-high_numbers_of_children_growing_up_homeless_in_temporary_accommodation<br><br>These also act as resources for readers to find more information.
**Methods**	
Is a copy of the interview guide available (as an appendix or table)? It would be helpful to see how the Social Ecological Model and social determinants of health informed the questions more explicitly.	Please find the interview guide attached as an appendix to this paper.

Given the recruitment strategy, it seems there is a strong possibility of selection bias based on a variety of factors including willingness to participate, stable phone line, language spoken at home. Could you clarify eligibility characteristics, anyone who declined or was unreachable and then further address this bias in the discussion section?<br><br>Reviewer 2 -<br>If possible, please provide response rate of the contacted and completed participants, example of questions asked to participants (are they structured or semi-structured interview?)<br>– Authors – Please note this request is address by the second paragraph in this section, and by providing the interview schedule, as noted in the above cells. In addition, the study design section of the methodology identifies '<u>Data for the study was collected via semi-structured telephone interviews</u>' noting that the request in bracket was addressed in the original manuscript.	Thank you for the inquiry, we have added the content below, to help clarify recruitment and eligibility:<br><br>Method – Participants section, first paragraph:<br><br>'The use of multiple agencies, communication approaches and social media to recruit participants helped ensure that a diverse range of people living in TA were recruited, and their lived experiences represented in the research. This was important as we were particularly interested in representing different types of TA as well as ethnic groups and different languages. To help facilitate this, where necessary, we provided support for participation by employing translation services and providing data vouchers to cover phone calls'.<br><br>Participants section, second paragraph:<br><br>'Amongst the narratives gathered, we are confident this diversity was well represented. Nonetheless, we acknowledge the likelihood of selection bias, which is an inherent challenge in research involving transient populations. Factors such as willingness to participate, stable telephone access, language barriers, and time constraints associated with caring for young children may have influenced, those who were able to take part. Moreover, we were unable to calculate precise recruitment or dropout rates for two key reasons. First, we relied on third-sector organisations for recruitment and to approach families within their remit, so exact numbers of families approached are unknown. There were different waves of recruitment by the third sector partners as well to reflect the transient nature of this group. Second, we only approached and interviewd those families that have given us permission to access them. This limitation is discussed in the manuscript as part of a broader reflection on the methodological challenges of engaging marginalised and underserved communities'.<br><br>Discussion – Limitation Section:<br><br>'Finally, while we have focused on maximising inclusivity within our recruitment strategy across England to represent diversity in temporary accommodation types, ethnic backgrounds, and languages spoken, we recognise that selection bias may still have influenced our findings. Families who participated were more likely to have had consistent

	telephone access, sufficient time to engage, and, in some cases, more support from close relatives or third-sector organisations. Therefore, more vulnerable families who were experiencing acute instability, language barriers that prevented them from reading or understanding recruitment materials, legal or safeguarding fears, or reluctance to engage with research may be under-represented. Such recruitment challenges and resultant biases are well-documented in studies involving transient populations (Booth, 1999) and are particularly salient in research with families experiencing homelessness in the UK. These constraints should be considered when interpreting the transferability of our findings'.
You mention the risk of participation, but weren't all interviews done via phone? Please clarify.	We added this sentence, in the third paragraph of the participant section, for clarification – 'Risks for participation were not limited to physical risks, as interviews took place via the phone; they also included possible distress from sharing stress-inducing details of experiences of living in TA and any impact on child health. We had procedures in place to mediate distress if and when it occurred and also directed all participants to free resources available to them to mediate any distress from sharing their stories with us'.
COVID-19 should be consistently capitalized throughout.	Done
For your analytical approach, was it inductive or deductive? You say data were analyzed thematically-was any particular qualitative approach used for this? Phenomenology, grounded theory etc? Please define and clarify with literature where possible.	We added the sentence below, at the start of the analytical approach section, to help with clarity:<br><br>'The research used an inductive, analytical approach in our thematic analysis of the data informed by the guidelines set out by Braun & Clarke (2019). The approach, set out by (Braun and& Clarke, (2019), which provided the flexibility and in-depth approach required to identify all important themes within the dataset'.<br><br>Please note in regard to a particular type of qualitative approach being used – the beginning of the method sections outlines that:<br><br>'The research used a qualitative descriptive design to explore the experiences of parents with children under 5 years, who lived in TA in England, during the COVID-19 pandemic (Doyle et al. 2020; Tenny et al. 2017). A qualitative approach was adopted by the research, as it allows deeper, more meaningful

	insights into participant experiences (Bradshaw et al. 2017).'<br><br>Which outlines the approach taken, and the references used to inform the research, hence we believe this topic has already been addressed. We also note that the research used:<br><br>'The Social Ecological Model (Golden et al 2015) theory was used as a guide in the development of the interview schedule, to help explore key Social Determinants such as housing quality, access to healthcare and education, and the safety of the environment.'<br><br>Which outlines the theoretical ideal underpinning the interview schedule design.
Can you comment on the interviewers positionality and how that may have impacted the responses? In other words, where the lived experiences between the interviewers and interviewees very different and how might that have impacted the rapport and responses.	Thank you for the inquiry, we have added the following content:<br><br>The position of the researcher can impact the research in a number of ways. Researcher (KF) who conducted interviews is noted as white, middle class, employed and a securely housed person with no lived experience of homelessness, and hence an outsider to those interviewed (Breen, 2007). They were also new to the subject area, and so had no preconceived biases or orientations based on previous experience with the sample group. Moreover, as many families taking part in the research where single parents with young children, who had been particularly isolated and suffering difficult experiences living in TA during COVID-19 restrictions, there was a noted value in talking to someone outside of their inner formal, and informal support circles. Hence, the research interview acted as a way of witnessing participants' challenging situations, including complaints about staff, healthcare workers and accommodation, as well as their resilience, enabling the parents to feel heard and for their perspective to be valued and included. In addition, there was a noted power imbalance, due to the incentive offered to people to participate in the research. Conducting phone interviews was one way in which participants could retain some control, as the researcher was not entering their space and they could end the interview at any point. Moreover, the researchers guaranteed that participants would receive their incentive, regardless of their level of involvement. This was also

	a way for participants to manage their own risk of harm in taking part.
**Results**	
The results of this study are at least 5 years old now. How can you relate these findings to long-term impacts or areas for future planning, rather than just historical data? In other words, is it possible to add more about its relevance in present-day.	We have added this sentence to the limitation section -<br>'While we recognise that the data in this paper is from 2021 and is a few years old now, we believe the results still hold relevance to present day context. For example, in the 5 years since the onset of the COVID-19 pandemic, homelessness rates for families with children continue to be on the rise with research consistently pointing to unsuitability of TA for supporting health and wellbeing of children, with one key determinant being the wellbeing and parenting capacity of their parents (Shelter 2025; Crisis 2024; Rosenthal et al., 2023)'.
Some of the results are specific to the temporary accommodations and some are specific to more general COVID restrictions. Is there a way you could separate these out and highlight these nuances a bit more? For example, one participant notes her child could not use the playground- I assume that is a COVID restriction, not a housing related barrier? Can you elaborate more on how these may intersect?	Please note, clarification on this subject did exist in text already. However, throughout the results section, some additional clarifying sentences have been added, to help make it clearer, when a result reflects either – TA; Covid-19 restrictions; or both. These additional sentences are not listed here as they exist throughout the results sections and would take up considerable space. Instead, they have been highlighted in yellow throughout the results section of the main manuscript, to help make them distinct. One example is given below:<br><br>'Similarly, participants noted that existing in a small space due to TA, and being restricted to that space due to COVID-19 restrictions, limited the space their child could play in and, moreover, because the family's many belongings were compacted into that small space, the area potentially became unsafe'
The results revealed 4 themes; access to service, mental health and well-being, and support service and network.<br>a. Some themes are overlapping with each other. I would recommend the author to revise the selected extracts those represent the theme clearly, or using 'bold' font to emphasize some keywords.	Thank you for your comment, however the three themes you have identified, are essentially the same as three we outline in our manuscript –<br>1. Access to services – Restricted access to necessities<br>2. Mental health and wellbeing – Impact on parent<br>3. Support service and network – Support<br>Which we believe are an accurate reflection of the wider data, and hence no changes have been made in regard to this comment.
With the combination of 'being a parent', 'living in TA' and 'during COVID-19' which is very unique. This current version, for me, some	As noted above, we have made it clearer within the results, when the discussion relates to COVID-19, temporary accommodation, or both. We have also noted in the results, that the impact of one factor, is

extract represents common experience of covid-19 (such as environmental restriction issues that most people experiencing this too, and social support), which I think will be better if the extracts has been revised.	often hard to separate from the other, for parents in living TA, which adds to the complexity of their lived experiences and parenting capacity. The intersectionality of identities (living in TA, parent, pandemic survivor) inform each other and constitute a unique blend of experiences, and not disntict categories that might be considered additive in their challenges. These identities inform and construct each other through cojoined experiences in TA that cannot be clinically dissected and should be considered, at least in part, as a unique whole.
**Discussion**	
Can you clarify or re-word this sentence-it's meaning is unclear to me: "For example, from the results of this research, a participant experienced delays in treatment due to a delayed in medical information being passed on, between different area"	This sentence has been re-worded in text, to:<br><br>"For example, due to the relocation of a family from one TA to another, one participant experienced delays in medical treatment as their information was not transferred in time between treatment facilities"
More literature is needed in the discussion section to tie these findings back to current evidence.<br><br>Reviewer 2 -<br>To me, the discussion looks like a summary of the result, and also, not much mentioned about parenting children under 5. It would provide more insights if the authors pick up some key issues and discuss in depth which links to key messages of the paper	We note at the start of the discussion that:<br><br>'This is one of only a handful of qualitative studies examining the impact of living through the COVID-19 restrictions in England, from the experiences of parents with children under 5, who also lived in TA during this period (Lakhanpaul et al 2023; Rosenthal et al. 2023). Thus, this study offers critical and unique insights into the impact of such events on these marginalised populations'.<br><br>Of the articles we draw on to inform the discussion of qualitative experiences of TA in our introduction (such as Lakhanpaul et al 2023; Rosenthal et al. 2023; Tu et al 2023; Parry et al., 2022; Taylor & Edwards 2012) – all are used in the discussion to help tie what results we have, to current literature on the topic. Later in the discussion, we start a paragraph with 'More broadly, the findings speak to…' to tie the results to a boarder set of literature, hence we believe the discussion adequality ties the results to current literature. However, we have also provided two additional discussion points, including –<br><br>1. 'Indeed, COVID-19 pandemic has introduced new challenges to families raising young children in TA, with closures to playgrounds and community centres and moving primary care into online or phone-based appointments. Some of these unique challenges are

	not relevant today, with playgrounds and community centres regaining their usual operations. However, some of the modifications in primary care are still faced by families today (like phone-based appointments)'.<br><br>2. 'A further point of intersection between the pandemic TA context and today, is the fact of its unsuitability for child health and development (Shelter 2025; Crisis 2024). While the pandemic exposed children to this environment for a considerably longer time due to lockdowns, children are still being placed in accommodation that is damp, unhygienic, overcrowded, and too cold (Shelter 2025; Crisis 2024). Therefore, negative impact of unsuitable housing on children and their parents have not disappeared with the pandemic, and remains a present challenge for around 150, 000 families across the UK'.<br><br>At the end of the discussion to help flesh the idea out.
**Limitations**	
While you note the limitations of this work, it would be helpful to see areas for future direction to continue and complement these findings as noted above.	Thank you for the suggestion, an additional section, titled 'Future Research', has been added to the end of the manuscript, to address this point, along with the paragraph:<br><br>'Future research should consider three key avenues. First, it is important to contextualise these findings within specific cultural groups and migration journeys, as these families are likely to share traumatic experiences unrelated to TA. They also come with the wealth of cultural knowledge, that can be harnessed in support of their parenting journeys in the UK. Second, children with complex needs also require focused exploration regarding parenting support and environmental risk in TA. Finally, exploring specific domains of parenting in more detail would uncover more learning within areas of sleep, nutrition, education and so forth'.
**Implication and impact**	
The results show strong evidence of how parents living in TA were suffered from COVID19. I would love to read some sentences from the authors suggesting future directions or strategies to improve quality of live in TA in England.	Thank you for this suggestion. We have already provided insights into future research avenues as a response to the reviewer above. We also include several points on how to improve parenting capacity for families raising young children in TA below:<br><br>To support parents' capacity to parent their children in resource limited settings like TA in England, it is important to dress three key limitations. First, child-

	centred accommodation is crucial to provide the environment within which children can thrive and parents can support their children's health and development. This means accommodation that is equipped with sufficient laundry, cooking, and food storage facilities, hygienic bathroom and toilet, capacity to cool and heat the space, absence of mould, space to crawl and walk, play space indoors and outdoors to name a few. Second, families need to be supported by an interconnected network of services (health, education, housing, social care). Siloed care leads to re-traumatisation of parents and many families falling through the cracks of a disjointed system. Finally, families need to be placed within networks of support, be that their existing networks, with specific help from link workers and health visitors who reach out to these families, or within accommodation with families (and not singles) that can provide information and help.

VERSION 2 - REVIEW

**Reviewer**	**1**
**Name**	**Schindler-Ruwisch, Jennifer**
**Affiliation**	**Fairfield University**
**Date**	**24-Sep-2025**
**COI**	

I find the revised paper much improved. The only remaining query I have relates to lines 36-51 in which bias and other limitations are discussed in the Methods section. This discussion should be moved to the Discussion (limitations) section as I feel it is more appropriate there to discuss methodological limitations than where it is placed now. Otherwise, I have no further comments. Thank you!

**Reviewer**	**2**
**Name**	**Wiwattanapantuwong, Juthatip**
**Affiliation**	**Chulalongkorn University**

Date **08-Oct-2025**

COI

The author has responded to all reviewer's comments.
